# Metagenomic and metabolomic analyses of fecal samples from civet-digested coffee in Vietnam

**DOI:** 10.7717/peerj.21262

**Published:** 2026-05-27

**Authors:** Tam Thi Thanh Tran, Oanh Thi Kieu Nguyen, Phuong Hanh Hoang, Nhung Phuong Nguyen, Huong Thi Mai To, Hoa Quynh Nguyen

**Affiliations:** University of Science and Technology of Hanoi, Vietnam Academy of Science and Technology, Hanoi, Vietnam

**Keywords:** Civet coffee, 16S rRNA metagenomics, Gut microbiota, Untargeted metabolomics, UPLC-QToF HRMS

## Abstract

**Background:**

Civet-digested coffee originates from the feces of civets that consume coffee cherries, where microbial fermentation in the gastrointestinal tract imparts distinctive flavor attributes, thereby enhancing its global reputation and market value. Gut microbiota is considered important drivers of coffee-bean fermentation, potentially shaping the unique and region-specific flavor characteristics of civet-digested coffee. To address this context, the present study integrated metagenomic and metabolomic analyses to compare the gut microbiota and secondary metabolites involved in coffee-bean fermentation inside Vietnamese civets.

**Methods:**

Fecal samples were collected under two dietary conditions: a standardized one containing 20% protein, 6% fiber, and 0.4–1.5% lysine, and the same diet supplemented with coffee cherries. Metagenomic 16S rRNA sequencing and untargeted ultra-performance liquid chromatography quadrupole time-of-flight (UPLC-QTOF) revealed clear differences between the two groups.

**Results:**

Integrated metagenomic and metabolomic analyses revealed clear distinctions between the two groups. Civets on the coffee-cherry diet exhibited higher microbial diversity at the family and genus levels. Specifically, among 31 classified bacterial genera showing a trend toward significant differences in abundance, *Enterococcus* and *Escherichia/Shigella* decreased, whereas *Gluconobacter*, and *Pseudomonas* increased following the diet shift. Metabolomic profiling identified 46 metabolites across both ionization modes, and strong correlations were observed between microbial genera and metabolite profiles. Specifically, 6-hydroxyangolensic acid methyl ester, 4-aminobenzoic acid and caffeine were more abundant in civets on a coffee-cherry diet, meanwhile the other nine metabolites were more prevalent in the normal diet. Overall, the findings demonstrate that civet gut microbiota and metabolic output were highly responsive to dietary inputs, and that coffee cherries promoted a unique fermentation environment. This represents the first integrative metagenomic and metabolomic study of civets consuming coffee in Vietnam, providing valuable insights into microbial contributions to coffee fermentation.

## Introduction

Civet-digested coffee or kopi luwak (hereafter referred to as civet coffee), is one of the most expensive coffees around the world. This coffee is produced through a natural fermentation process occuring in the digestive system of the Asian palm civet (*Paradoxurus hermaphroditus*). Upon consumption of ripe coffee cherries, the pulp is digested and absorbed, while the beans remain largely intact and are excreted in the feces. During gastrointestinal transit, the beans are exposed to a complex mixture of digestive enzymes and gut microbiota, resulting in partial fermentation. This exposure leads to significant biochemical transformations in the beans, including the breakdown of proteins, sugars, and polysaccharides, which ultimately alter the sensory properties of the coffee (Marcone, 2004; Haile & Kang, 2019). Since its discovery in the 19th century, the high demand for civet coffee has continued to grow, which has led to a rampant increase in its production. Nowadays, the high price of kopi luwak reflects its exclusivity and unique digestive fermentation process (Freitas et al., 2024).

Numerous studies have reported distinct physicochemical and sensory differences between civet and normal coffee. These differences are generally attributed to the unique fermentation process that occurs within the civet’s gastrointestinal tract (Chan & Garcia, 2011). Civet-digested beans exhibit altered levels of key compounds, including reduced acidity and bitterness, which are often linked to lower concentrations of chlorogenic acids and modified caffeine content (Muzaifa, Hasni & Rahmi, 2019). For example, civet coffee tends to have slightly lower caffeine levels compared to regular beans post-roasting, possibly due to partial degradation during digestion and minimal losses during roasting (Muzaifa et al., 2020). Metabolomic profiling further indicates elevated levels of organic acids (*e.g*., malic acid and citric acid), along with increased concentrations of trigonelline and certain lipid components. Together, these changes contribute to a complex flavor profile characterized by earthy, nutty, and chocolaty notes (Jumhawan et al., 2013). Structural analyses using scanning electron microscopy have demonstrated micro-pitting on the surface of civet coffee beans. This may result as a consequence of enzymatic activity during digestion, in contrast to the smoother surface of normal coffee beans (Chan & Garcia, 2011). Furthermore, civet coffee has been associated with a richer aroma, attributed to a higher abundance of volatile compounds, including furans, pyrazines, and pyridines (Muzaifa et al., 2020). These chemical and physical transformations suggest that the fermentation process inside the civet’s digestive system plays a pivotal role in reshaping the metabolite landscape of the coffee beans, which ultimately contributes to their distinctive sensory properties.

Recent advances in omics technologies have encouraged investigation of the fermentation processes occurring in the civet gastrointestinal tract during coffee cherry consumption. Metagenomic analyses of civet fecal samples have reported a predominance of *Gluconobacter* spp., which harbor genes associated with sulfur amino acid metabolism, caffeine degradation, and other pathways relevant to fermentation (Watanabe et al., 2020). These microorganisms are therefore thought to contribute to biochemical transformations of coffee beans as they pass through the intestine. In contrast, Winarsih et al. (2025) found that civets fed robusta coffee cherries harbored gut communities dominated by *Escherichia coli*, lactic acid bacteria, and yeasts. These author further suggested that host species and sex may influence microbial diversity, although the sample size was limited. A subsequent study integrating metagenomics and metabolomics (Vale et al., 2024) primarily focused on microbial contributions to coffee flavor development rather than intestinal processes. Most metabolomic investigations have also been conducted on civet coffee beans themselves (Farag et al., 2023; Febrina, Happyana & Syah, 2021, 2023; Jumhawan et al., 2013; Noel, Lebrilla & Garcia, 2016; Sari, Wahyudi & Sulihkanti, 2012) rather than on civet fecal material. Consequently, a clear gap remains in integrative multi-omics studies directly linking microbial activity to specific metabolite changes in civet feces. Combining metagenomic and metabolomic approaches would provide a more comprehensive understanding of fermentation mechanisms within the civet gut. It may also support the development of biomimetic fermentation strategies as well as improved authentication methods for civet coffee.

In addition, a major challenge in these omics-based studies is the considerable variability among findings. Civets live in diverse geographic environments, and their diets vary depending on farming practices, which contributes to the unique flavor characteristics of civet-digested coffee. Furthermore, diet is a major factor influencing the composition and diversity of the gut microbiota in mammals (Albenberg & Wu, 2014; Huang et al., 2025; Li et al., 2022; Ross et al., 2024; Wu et al., 2011), with different dietary patterns and feeding guilds giving rise to distinct microbial communities. In addition, certain dietary components, such as coffee, have been linked to increased microbial diversity and a higher abundance of beneficial bacterial taxa in human models (Asnicar et al., 2021; Saygili, Hegde & Shi, 2024). These factors together influence the composition of the civet gut microbiome, leading to substantial variation across studies. To address these knowledge gaps, the present pilot study integrates 16S rRNA-based metagenomic sequencing with untargeted ultra-performance liquid chromatography quadrupole time-of-flight (UPLC-QTOF) metabolomics to characterize fecal samples from civets under two dietary conditions: a standard diet and the same diet supplemented with coffee cherries. By jointly analyzing microbial diversity, taxonomic composition, metabolite profiles, and their correlations, we aim to (i) determine how coffee consumption reshapes the civet gut microbiome, (ii) identify diet-associated metabolites potentially linked to fermentation, and (iii) uncover functional relationships between specific taxa and chemical transformations.

Studying fermentation within the civet gastrointestinal tract presents several inherent biological and methodological challenges. Unlike controlled *in vitro* systems, *in vivo* fermentation is likely influenced by host-specific factors such as gut transit time, digestive enzyme activity, immune responses, bile acid composition, and gastrointestinal microbiota structure. Such factors may introduce substantial inter-individual variability and limit precise control over fermentation parameters. Moreover, ethical constraints restrict experimental manipulation and large-scale sampling, making it difficult to directly attribute specific metabolite transformations to defined microbial taxa within this complex ecosystem. To partially address these constraints, the present pilot study employed a paired within-subject design in which the same nine female civets were sampled under two defined dietary regimens within a single standardized farming system. By maintaining consistent husbandry conditions and introducing a controlled dietary shift (standard diet *vs* coffee-cherry supplementation), environmental and host-related confounding factors were minimized. Furthermore, the integration of 16S rRNA metagenomics with untargeted UPLC–QTOF metabolomics enabled simultaneous characterization of microbial community shifts and associated metabolic outputs. Such approach thereby strengthens functional inference within a physiologically relevant *in vivo* context. Overall, this integrative framework is of great importance in providing mechanistic insight into civet coffee fermentation in Vietnam.

## Materials AND methods

### Sample collection

Civet fecal samples used in this study were sourced from Kien Cuong Civet Coffee Company in Buon Ma Thuot City, Dak Lak Province, Vietnam, which operates under business registration certificate No. 6000771878 (issued on June 14, 2021, by the Department of Planning and Investment of Dak Lak Province, Vietnam). The care and management of palm civets at the company are conducted in cooperation with the Dak Lak Provincial Forest Protection Department, Vietnam. Moreover, the company was granted a Useful Solution Patent by the National Office of Intellectual Property on the invention “Method of producing weasel coffee” in 2012. Throughout the study, no experimental interventions were performed on the civets; instead, only the company’s routine husbandry practices were observed and followed.

Samples were collected twice in April 2024 from nine female Asian palm civets (*Paradoxurus hermaphroditus*). The civets were fed once daily between 17:00–18:00 with a standard diet consisting of 150 g of food containing 20% protein, 6% fiber, and 0.4–1.5% lysine. Dietary protein is primarily supplied by chicken, while fruits including bananas, jackfruit, and papaya serve as important sources of fiber. During the coffee season, coffee cherries were incorporated into their diet every other day. The *Coffea arabica* coffee cherries supplied for civets were also planted in Kien Cuong Civet coffee fields in Buon Me Thuot city, DakLak Province (108.02935° longitude, 12.677369° latitude, 435-m altitude). Only ripe cherries were chosen as the food for the civets. The first fecal sampling was carried out when the civets were fed with coffee cherries added to their diet (hereafter referred to as coffee-cherry diet), whereas the second sampling was conducted one week later, when the animals were maintained solely on the standard diet (hereafter referred to as normal diet). Feces were recuperated the following morning after the civets were fed with each type of diet as their latest meal, placed in separate plastic bags inside a container with dry ice, and transported by air to the University of Science and Technology of Hanoi (Hanoi, Vietnam) on the same day. All samples were stored at −80 °C until DNA extraction.

### Metagenome analysis

#### Genomic DNA extraction

DNA isolation was performed on civet’s fecal samples without coffee beans using the QIAamp PowerFecal Pro DNA Kit (Qiagen, Hilden, Germany), following the manufacturer’s guidelines. Prior to DNA extraction, fecal samples were thawed and homogenized thoroughly. Homogenization process was performed twice at 25 Hz for 5 min per cycle using a Qiagen TissueLyser II (Qiagen, Hilden, Germany). The quality and purity of the extracted DNA were assessed using a Thermo Scientific NanoDrop 2000/2000c Spectrophotometer (Thermo Fisher Scientific, Waltham, MA, USA). Samples with an OD_260/280_ ratio between 1.8 to 2.0 were considered of high DNA purity and subsequently processed for sequencing.

#### 16S rRNA metagenomic sequencing

To compare the bacterial microbiome in civet’s feces under different diets, 16S rRNA metagenomic sequencing was employed. The 16S gene, approximately 1,500-base pairs (bp) in length, is present in all prokaryotic genomes and provide insights into the microbial composition, abundance, and potential functional roles of the bacteria in the civets’ digestive systems through comparison with public database (Kim & Chun, 2014).

DNA samples were sequenced at Genewiz, Inc. (South Plainfield, NJ, USA) using a MetaVX Library Preparation Kit to create a sequencing library. The V3–V4 region of the 16S rRNA gene was amplified using the forward primer 5′-CCTACGGRRBGCASCAGKVRVGAAT-3′ and the reverse primer 5′-GGACTACNVGGGTWTCTAATCC-3′. Then, polymerase chain reaction (PCR) was conducted using a mixture of 20 ng template DNA, 1 µL of each primer, 2.5 µL of TransStart buffer, 2 µL of dNTPs, and 0.5 µL of TransStart Taq DNA polymerase (TransGen Biotech Co., Ltd., Beijing, China). The amplification procedure was initiated with 3 min of denaturation at 94 °C, followed by 24 cycles of 5 s at 95 °C, 90 s of annealing at 57 °C, 10 s of elongation at 72 °C, and terminated by 5-min extension at 72 °C. Libraries were purified using magnetic beads, and paired-end sequencing was conducted on an Illumina Miseq (Illumina, San Diego, CA, USA).

#### Bioinformatics analysis

16S rRNA sequencing data were processed using QIIME 2 v.2024.5 (Bolyen et al., 2019). First, paired-end reads were imported into QIIME2, and low-quality sequences and primers were trimmed using cutadapt (Martin, 2011) integrated within QIIME2. Next, reads were denoised, dereplicated, and filtered for chimeras using qiime dada2 denoise-paired. Amplicon sequence variant (ASV) representative sequences were taxonomically classified against the SILVA v.138 reference database at 99% similarity using qiime feature-classifier (Quast et al., 2012). A phylogenetic tree was constructed from the representative sequences using qiime phylogeny align-to-tree-mafft-fasttree. The number of quality-filtered reads with assigned taxonomy ranged from 110,744 to 301,545 per sample (Table S1). Rarefaction curves were subsequently produced using the vegan package’s rarecurve function based on the ASV table (Fig. S1). Alpha and beta metrices were calculated using qiime diversity core-metrics-phylogenetic with a sampling depth of 110,744, which as the minimum read count observed across all samples.

### Metabolome analysis

#### Chemical preparation

High performance liquid chromatography-grade methanol, acetonitrile (>99.5% purity), formic acid (FA, >98% purity) and camphorsulfonic acid were purchased from Sigma–Aldrich (St. Louis, Missouri, MO, USA). Ultra-pure water was collected from a Milli-Q Integral 3 water purification system (Merch, Darmstadt, Germany) and used for the preparation of standards, extraction solutions and mobile phase solutions.

#### Metabolite extraction

Fifty milligrams of each fecal sample without coffee beans were dissolved in 1 mL of 80% methanol containing 3.25 ppm of 10-camphorsulfonic acid, followed by ultrasonic sonication for 15 min. Samples were then incubated at 65 °C for 45 min and centrifuged at 14,000×*g* for 10 min. The resulting supernatants were filtered through 0.22-µm nylon syringe filters prior to analysis by an ultra-performance liquid chromatography with a quadrupole time-of-flight (UPLC-QToF) system.

#### UPLC-QTOF analysis

Metabolite analysis was performed using an ACQUITY UPLC I-Class Plus system coupled to a Xevo G3 ESI/QTOF high-resolution mass spectrometer (Waters Corporation, Milford, MA, USA). Chromatographic separation was conducted on an ACQUITY UPLC BEH C18 (1.7 µm, 2.1 × 50 mm, 130 Å) using a composition of solvent A (H_2_O with 0.1% FA) and solvent B (acetonitrile with 0.1% FA) as the mobile phase. The gradient program was as follows: 20% B for 2 min, ramped to 95% B over 10 min, held at 95% B for 5 min, decreased to 20% B over 3 min, and re-equilibrated for 2 min. The flow rate was set at 0.3 mL/min, with an injection volume of 5 µL.

The QToF parameters were set up under MSe continuum mode which scanned from m/z of 100 to 1,500 with a scan time of 0.1 s. In low-energy mode, a cone voltage of 6 V was applied, while in a high-energy mode, a voltage ramp from 15 to 40 V was applied. Besides, the capillary voltage of 3.00 kV (positive mode), source temperature of 120 °C, desolvation temperature of 450 °C, cone gas of 30 L/h, and de-solvation gas of 950 L/h were set for electrospray ionization quadrupole time of flight mass spectrometry (ESI-QTOF-MS).

#### Data preprocessing

Raw data files were converted to the ABF format using the Analysis Base File Converter and subsequently processed with MS-DIAL (version 5.5.250627). The MS1 and MS2 tolerances were set at 0.01 and 0.025 Da, respectively. Peaks with a height greater than 500 were extracted using a mass slice width of 0.1 Da and smoothed with a linear weighted moving average. Spectrum deconvolution was performed with a sigma window value of 0.5, while other parameters were retained as default. Metabolites and lipids were annotated against the FAHFA, Fiehn HILIC, LipidBlast 2022, and LC-MS/MS Positive Mode spectral libraries (https://mona.fiehnlab.ucdavis.edu/), applying MS1 and MS2 tolerances of 0.04 Da. Annotation included all possible adduct ion species. Peak alignment was conducted with a retention time tolerance of 0.1 min and MS1 tolerance of 0.015 Da. To eliminate background signals, features with a sample maximum-to-blank average ratio <5 were removed.

#### Statistical analysis

Differences in alpha diversity, bacterial taxa and metabolites between the coffee-cherry and normal diets were assessed using the Wilcoxon signed-rank test. *P*-values were adjusted for multiple testing using the Benjamini–Hochberg method to control the false discovery rate (FDR), with the significance threshold of 0.05. Beta diversity, based on Weighted and Unweighted UniFrac distances, was visualized using principal coordinate analysis (PCoA), and differences were evaluated *via* permutation multivariate analysis of variance (PERMANOVA). Correlations between metagenomic and metabolomic profiles were assessed through coinertia analysis implemented in the ade4 R package, with significance determined using a Monte Carlo permutation test (RV.rtest function). The Spearman correlation method was applied to assess the associations between bacterial taxonomic abundances and metabolite concentrations.

For multivariate metabolome analyses, the positive and negative ion datasets were combined and scaled prior to analysis. Principal component analysis (PCA) was employed as an unsupervised classification method, while partial least squares discriminant analysis (PLS-DA) served as a supervised approach to distinguish between the two dietary groups. Model validation was conducted using leave-one-out cross-validation. Wilcoxon signed-rank test was used for comparison of metabolites between the coffee-cherry diet and the normal diet of the Asian palm civets in this study. Features with strong associations, defined as those showing an absolute Spearman’s rho greater than 0.7, and statistical significance, were visualized using pheatmap R package v.1.0.13. All analyses were performed using R version 4.2.1 (R Core Team, 2021).

## Results

### Metagenome analysis

#### Diversity of gut microbiota in Asian palm civets

**Figure 1:**
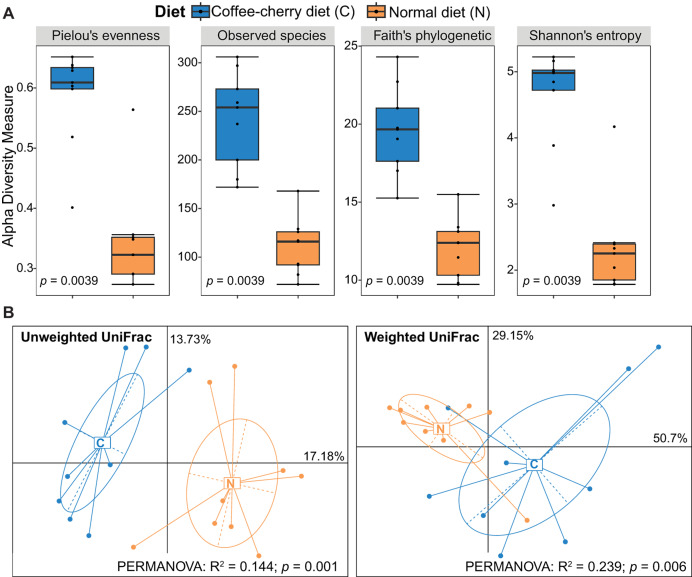
Comparison of alpha and beta diversity of gut microbiota between Asian palm civets with a diet consisting of coffee cherries and those with a normal diet. (A) Boxplots represent alpha diversity indices. *P* values were calculated using Wilcoxon signed-rank tests for paired samples. (B) Principal Coordinate Analysis illustrates beta diversity between the two diets based on unweighted and weighted UniFrac distances. Permutational multivariate analysis of variance indicated that both models were statistically significant.

The gut microbiota of Asian palm civets consuming coffee cherries were compared to that of civets on a normal diet. Overall, these two diets displayed a significant impact on both alpha and beta diversity on the fecal bacterial communities. Specifically, Pielou’s evenness scores, a measure of species equitability within a community ranging from 0 to 1, were 0.59 ± 0.08 for the gut microbiota under the coffee-cherry diet and 0.34 ± 0.09 under the normal diet. The total observed ASVs (estimated species richness) were 242 ± 48.8 and 110.56 ± 29.38, Faith’s phylogenetic diversity indices were 19.6 ± 2.82 and 12.01 ± 1.9, and Shannon’s entropy indices were 4.65 ± 0.74 and 2.34 ± 0.73 for the coffee-cherry diet and the normal diet, respectively. Faith’s phylogenetic diversity reflects the evolutionary breadth within a community, whereas Shannon’s entropy measures both species richness and evenness. Together, these indices describe microbial community structure. All four alpha diversity indices were significantly higher in the coffee-cherry diet group than in the normal diet group (Wilcoxon signed-rank tests, *p* < 0.05) (Fig. 1A).

For the quantification of the between-group dissimilarity in microbial composition, both weighted and unweighted Unifrac distances were used for the assessment of beta diversity. The PCoA plots reveal a clear separation of the gut microbiota between the two diets (Fig. 1B). Although minor overlap was observed in the weighted Unifrac analysis, the unweighted Unifrac distance suggested a distinct delimitation between the two groups. Furthermore, a significant difference was also observed using PERMANOVA tests (Unweighted Unifrac: *R*^2^ = 0.144, *p* = 0.001; Weighted Unifrac: *R*^2^ = 0.239, *p* = 0.006). These results indicate that the gut microbiota of the civets consuming a coffee-cherry diet differed substantially from that of the civets on a normal diet.

#### Taxonomic compositional changes in Asian palm civets associated with diet

A detailed examination of bacterial taxonomy from phylum to genus would provide a deeper insight into the gut microbiota of civets under different diets. At the phylum level, Proteobacteria, Firmicutes, Actinobacteriota, Bacteroidota, and Fusobacteriota were the most prevalent ones. Notably, Proteobacteria exceeded 50% relative abundance in 13 of total 18 samples (Fig. S2A). Although the ratio of Proteobacteria to Firmicutes was approximately twice as high in civets on a normal diet (3.01 ± 1.66) compared to those fed coffee cherries (1.49 ± 0.98), this difference was marginally statistically significant (Wilcoxon signed-rank tests, *p* = 0.055) (Fig. S2D). At the family level, coffee-cherry diet exhibited a more diverse and heterogeneous bacterial composition, whereas normal diet was predominantly composed of Enterococcaceae and Enterobacteriaceae families (Fig. S2B). Similarly, genus-level pattern was consistent with the family-level trends, indicating a remarkable shift in the gut microbiome according to diet (Fig. S2D). Specifically, a distinct difference was observed not only in the number but also in the relative abundance of genera between the two groups. *Ligilactobacillus* and *Streptococcus* were ubiquitous in the coffee-cherry diet group but largely absent in the normal diet group. In contrast, *Enterococcus* dominated in the normal diet samples but only constituted minor portions in the coffee-cherry diet samples.

**Figure 2:**
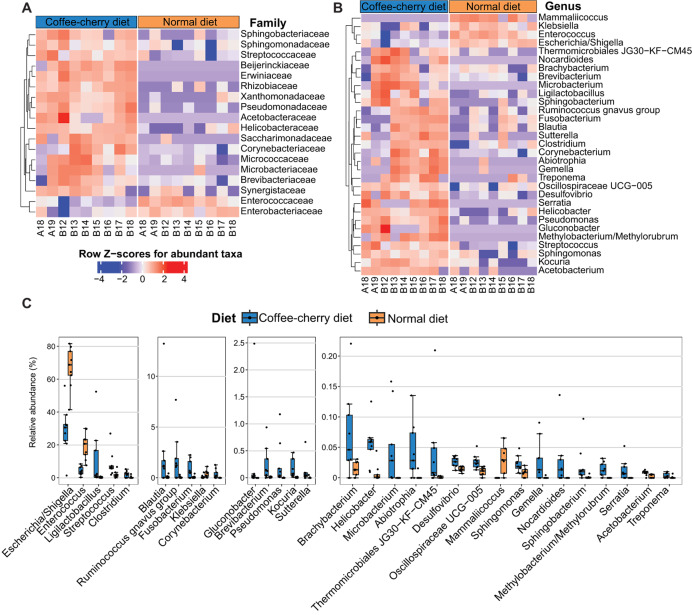
Comparison of bacterial taxa of gut microbiota between Asian palm civets with a diet consisting of coffee cherries and those with a normal diet. Heatmaps show taxa at the family level (A) and at the genus level (B) that exhibit significant differences between the two diet groups (nominal *p* < 0.05 and adjusted *p* < 0.1). The color bars at the top of the heatmaps indicate the grouping of civet cats based on their dietary regimens. (C) Boxplots illustrate the relative abundance of genera that show significant differences between the two groups. *P* values were calculated using Wilcoxon signed-rank tests for paired samples and adjusted to control the false discovery rate with the Benjamini–Hochberg method. Only classified taxa are shown.

A total of 31 families showed significant differences in relative abundance between the two diet groups (nominal *p* < 0.05) (Table S2). Among these, 18 classified families showed marginal significance after the FDR adjustment (adjusted *p* < 0.1). Sixteen families, including Sphingomonadaceae, Sphingobacteriaceae, Streptococcaceae, Helicobacteraceae, Pseudomonadaceae, Xanthomonadaceae, Acetobacteraceae, Erwiniaceae, Beijerinckiaceae, Rhizobiaceae, Synergistaceae, Brevibacteriaceae, Microbacteriaceae, Micrococcaceae, Corynebacteriaceae, and Saccharimonadaceae, were enriched in the coffee-cherry diet samples, whereas Enterococcaceae and Enterobacteriaceae families were mostly found in normal-diet samples (Fig. 2A).

At the genus level, significant between-group variation was observed in 38 genera, of which 31 genera were classified (adjusted *p* < 0.1) (Table S2, Figs. 2B and 2C). Coffee-cherry consumption was associated with a higher abundance of 27 genera such as *Ligilactobacillus*, *Clostridium*, *Blautia*, *Gluconobacter*, *Ruminococcus gnavus* group, *Fusobacterium*, *Klebsiella*, *Corynebacterium, Brevibacterium, Pseudomonas, Kocuria, Sutterella, *etc**. Meanwhile, the normal diet group displayed higher number of *Escherichia*-*Shigella*, *Enterococcus*, *Klebsiella*, and *Mammaliicoccus*. Overall, these findings highlight substantial diet-associated shifts in the gut microbiota of Asian palm civets, with certain bacterial genera being highly abundant in one diet but nearly absent in the other. This underscores the impact of dietary composition on the gut microbial community structure in civet feces.

#### Metabolome analysis

**Figure 3:**
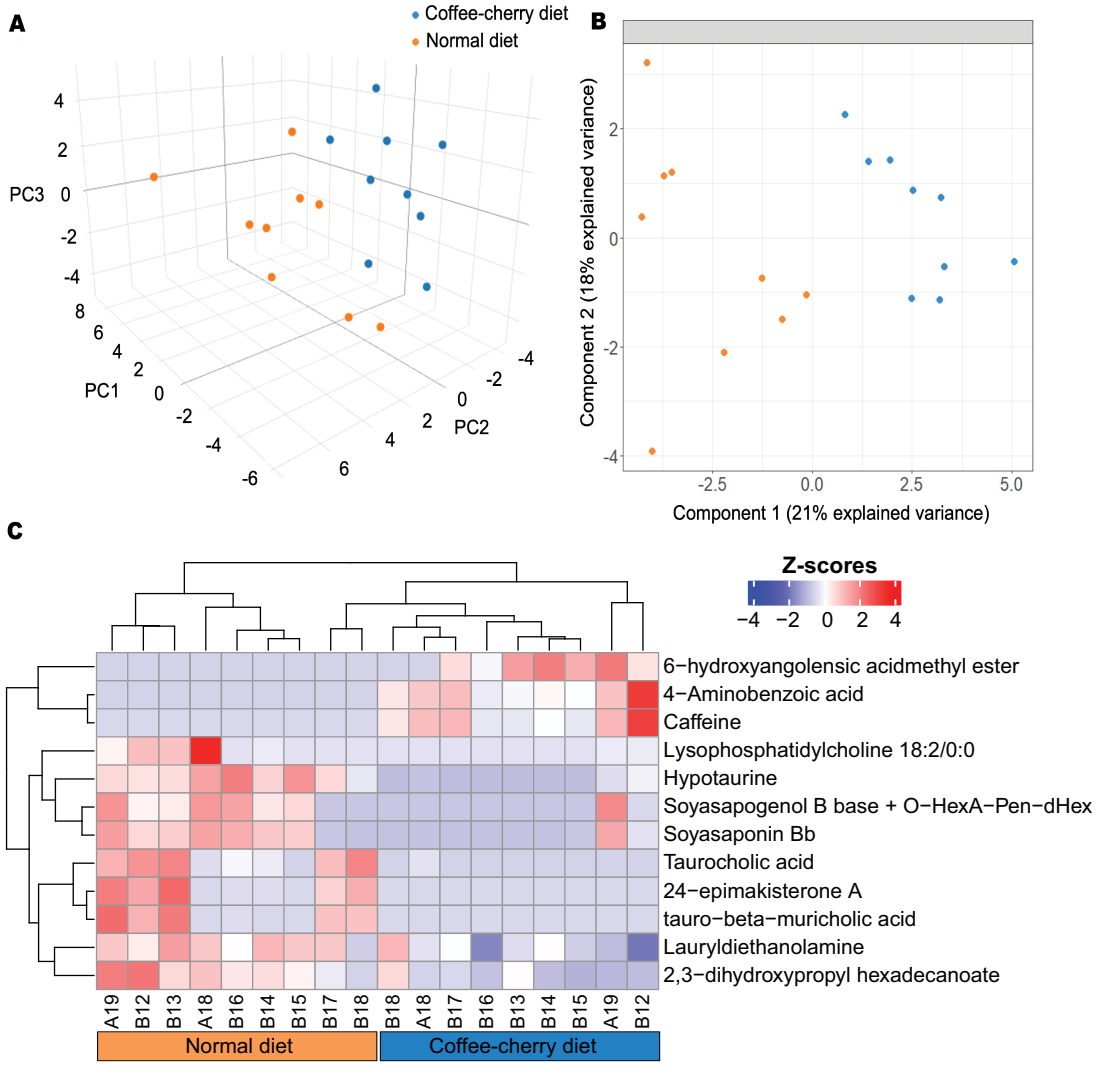
Comparison of fecal metabolites between Asian palm civets with a diet consisting of coffee cherries and those with a normal diet. (A) Principal component analysis using the first three principal components. (B) Partial least square-discriminant analysis using the first two components. (C) Heatmap showing fecal metabolites with significant differences between the two groups (nominal *p* < 0.05). Colors represent row-wise Z-scores of metabolite abundances, with red indicating higher abundance and blue indicating lower abundance. The color bars at the bottom of the heatmap indicate the grouping of civet cats based on their dietary regimens.

A total of 46 metabolite peaks were detected in fecal samples of civets under both positive and negative ionization modes across the two dietary groups (Table S3). Both unsupervised and supervised multivariate analyses revealed clear differentiation between the metabolomic profiles associated with the coffee-cherry and normal diets (Fig. 3). Principal component analysis (PCA) indicated partial separation along the first three principal components, which together accounted for 61% of the total variance (Fig. 3A). Meanwhile, partial least squares discriminant analysis (PLS-DA) provided a more distinct separation of the dietary groups, with the first three components explaining 49% of the total variance (Fig. 3B). Despite the slightly lower variance captured by PLS-DA, its supervised nature allowed for enhanced discrimination between groups, highlighting its suitability for identifying diet-associated metabolic biomarkers. Furthermore, leave-one-out cross-validation of the first three PLS-DA components yielded an accuracy of 99.5%, assessed on both the balanced error rate and overall error rate.

Among the detected metabolites, 12 differed significantly between the two dietary regimens (nominal *p* < 0.05) (Table S3 and Fig. 3C). 6-Hydroxyangolensic acid methyl ester, 4-aminobenzoic acid and caffeine were more abundant in Asian palm civets on a coffee-cherry diet. In contrast, LPC 18:2/0:0, Hypotaurine, Soyasapogenol B base + O−HexA−Pen−dHex, Soyasaponin Bb, Taurocholic acid, 24-epimakisterone A, tauro-beta-muricholic acid, Lauryldiethanolamine, 2,3-dihydroxypropyl hexadecanoate were more abundant in civets on a normal diet (Fig. 3C). However, these differences were no longer statistically significant after Benjamini–Hochberg correction (Table S4).

### Associations between the gut microbiota and fecal metabolite profiles

**Figure 4:**
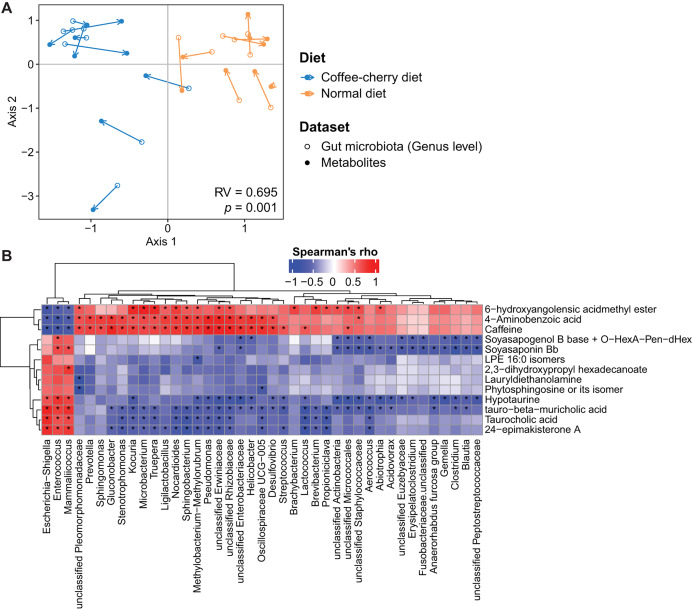
Correlation between the gut microbiota at genus level and fecal metabolite profiles in Asian palm civets. (A) Co-inertia analysis conducted to assess the relationship between bacterial genera (solid circles) and fecal metabolite profiles (empty circles). Colors represent civets fed exclusively on coffee cherries (blue) and those on a normal diet (yellow). Arrow lengths indicate the divergence between the two datasets. The RV coefficient between the datasets was calculated using a Monte Carlo permutation test. (B) Heatmap showing Spearman’s correlation between bacterial genera and fecal metabolites. Correlations were calculated using the Spearman correlation coefficient (Spearman’s rho). Only significant taxa or metabolites with a Spearman’s rho greater than 0.7 are displayed. Asterisks (*) indicate statistically significant correlations (*p* < 0.05).

The relationship between gut microbiota at the genus level and fecal metabolite profiles was evaluated using co-inertia analysis. A high overall similarity was observed between bacterial genera and metabolite profiles (Monte Carlo permutation test: RV = 0.695, *p* = 0.01) (Fig. 4A). Spearman correlation analyses revealed that the three metabolites, 6-hydroxyangolensic acid methyl ester, 4-aminobenzoic acid, and caffeine, showed strong negative correlation with *Escherichia-Shigella*, *Enterococcus*, and *Mammaliicoccus* (Spearman’s ρ < −0.7, adjusted *p* > 0.05), but strong positive correlations with several other genera (Spearman’s ρ > 0.7, adjusted *p* > 0.05). On the other hand, metabolites including Soyasapogenol B base + O−HexA−Pen−dHex, Soyasaponin Bb, 1-Palmitoyl-2-hydroxy-sn-glycero-3-phospho-ethanolamine (LPE 16:0) isomers, lauryldiethanolamine, phytosphingosine (or its isomer), hypotaurine, tauro-β-muricholic acid, taurocholic acid, and 24-epimakisterone A showed the opposite trend (Table S5 and Fig. 4B). These results indicate that specific fecal metabolites were closely associated with diet-driven variations in the civet gut microbiota.

## Discussion

This study provides the first integrated metagenomic–metabolomic characterization of fecal samples from Vietnamese civets consuming coffee cherries and directly links gut microbial shifts to changes in fermentation-associated metabolites. While previous investigations have independently described civet gut microbiota or chemical differences in kopi luwak beans, few studies have simultaneously examined microbial communities and metabolite outputs within the same biological system. By combining 16S rRNA profiling with untargeted UPLC–QTOF metabolomics, our results facilitate comprehensive insight into how diet-driven microbial processes may shape the chemical signature of civet-digested coffee.

Coffee-cherry consumption significantly increased alpha diversity and phylogenetic richness compared with the standard diet, indicating that the addition of plant-derived substrates creates a more heterogeneous ecological niche. This increased diversity was likely due to the addition on fiber, polyphenols, antioxidants, and other nutrients provided by the coffee cherries. In total, 27 classified bacterial genera were found to be enriched in of coffee cherry diet, with *Ligilactobacillus*, *Streptococcus*, *Clostridium*, *Blautia, Fusobacterium, Corynebacterium*, *Gluconobacter*, *Brevibacterium, Pseudomonas*, *Kocuria, Sutterella, Helicobacter, Abiotrophia, Desulfovibrio, Gemella* and *Acetobacterium* among the most notable taxa. Recent studies have discovered hundreds of microbial species involved in coffee fermentation (de Melo Pereira et al., 2014; Silva et al., 2013) with *Enterococcus* and *Enterobacteriaceae* bacteria among the key taxa contributing to fermentation in industrial settings (Elhalis, Cox & Zhao, 2023). Several of the bacterial genera identified in this study have also been implicated in animal digestion. Suhandono et al. (2016) identified *Bacillus, Pseudomonas, Pantoea, Escherichia, Lactobacillus*, and *Kocuria* from bacterial isolates cultured from the entire gastrointestinal tract of a in wild civets in Indonesia. Previous research has reported the genus *Gluconobacter* as abundant in Asian palm civet feces, suggested that its genes involved in hydrogen sulfide and sulfur-containing amino acid metabolism may be critical in the fermentation process of kopi luwak beans and flavor development (Agyirifo et al., 2019; Watanabe et al., 2020). The presence of lactic acid bacteria (LAB) including *Ligilactobacillus*, *Streptococcus*, *Abiotrophia*, and *Gemella* was also detected in this study. LAB comprises a group of bacteria commonly found in the gastrointestinal and genitourinary tracts of animals and are well-known for their health-promoting functions, including immunomodulation, maintenance of intestinal integrity, and resistance to pathogens (Marcone, 2004; Muzaifa, Hasni & Rahmi, 2019). For instance, increased abundance of *Ligilactobacillus* may positively influence intestinal health, as recent studies highlight its probiotic potential and ability to modulate intestinal immunity (Chuandong et al., 2024; Huang et al., 2016). Analysis of excreta from several civet species have identified dominant microorganisms that may contribute to caffeine metabolism (Muzaifa, Hasni & Rahmi, 2019; Winarsih et al., 2025), thereby potentially enhancing the sensory quality of coffee brewing (de Jesus Cassimiro et al., 2022). Alongside with LAB, an enrichment of several non-LAB taxa was observed. Notably, *Pseudomonas* species, known to degrade monoterpenes and diterpene acids, phytotoxins present in coffee beans (Zhang et al., 2019; Zhao, Lin & Guo, 2022), may contribute to mitigating coffee cherry toxicity in civets. Furthermore, *Pseudomonas* strains have been reported to degrade caffeine (Asano, Komeda & Yamada, 1993; Hakil et al., 1998; Yamaoka-Yano & Mazzafera, 1999; Woolfolk, 1975), and this genus was found to be marginally more abundant in civets fed on a coffee-cherry diet compared to those on a normal diet (Fig. 2C). The enrichment of this genus in coffee-fed civets therefore provides a plausible mechanistic explanation for the transformation of caffeine and related alkaloids during digestion.

Civets consuming coffee-cherry diet also exhibited an overrepresentation of several potentially pathogenic genera such as *Fusobacterium, Helicobacter, Pseudomonas, Desulfovibrio* and *Clostridium*, raising concerns about host health and the potential transmission of pathogenic bacteria to humans through civet coffee processing. Indeed, the genus *Helicobacter*, particularly *Helicobacter pylori*, possesses multiple virulent genes and pathogenic islands, which are well studied in relation to gastric diseases in humans (Šterbenc et al., 2019). *Desulfovibrio* and *Clostridium* species have been reported to be associated with inflammatory bowel disease (Singh, Carroll-Portillo & Lin, 2023; Rodemann et al., 2007), whereas *Fusobacterium* has been recognized for its association with colorectal adenomas (McCoy et al., 2013). Although *Pseudomonas* plays an important role in digesting coffee cherries, certain species, including *Pseudomonas aeruginosa* are recognized as opportunistic pathogens, which is associated with nosocomial infections (Jangra, Sharma & Chhillar, 2022). The enrichment of the 27 genera was accompanied by a reduction in 4 genera, including *Mammaliicoccus, Klebsiella, Enterococcus*, and *Escherichia/Shigella*, in civets consuming coffee cherries. *Enterococcus* and *Escherichia/Shigella* have been reported as common commensal members of the gastrointestinal tract in poultry and wild animals such as the red panda (Khasanah et al., 2024; Zeng et al., 2018). However, the increased abundance of these two genera was found to be associated with diarrhea in piglets (Gryaznova et al., 2022). Among the four reduced genera, E*scherichia/Shigella, Enterococcus*, and *Mammaliicoccus* exhibited strong negative correlations with 6-hydroxyangolensic acid methyl ester, 4-aminobenzoic acid, and caffeine, suggesting potential interactions between host diet, gut microbiota profile, and fecal metabolites.

Beyond the gut microbiome, many of the same bacterial taxa are also reported in natural coffee fermentation processes. *Gluconobacter* is common both in civet gut communities and in naturally fermented coffee (International Coffee Organization, 2023; Lozupone et al., 2007). Other taxa, such as *Tatumella*, *Pantoea*, and *Enterobacter*, have been identified in anaerobic coffee fermentation (Lee et al., 2023), while *Erwinia herbicola* and *Klebsiella pneumoniae* are frequently associated with *Coffea arabica* fermentation (Avallone et al., 2002). Moreover, *Enterococcus* strains are present in fresh coffee cherries (Leong et al., 2014). Despite these findings, the functional roles of many genera in both animal digestion and coffee fermentation remain poorly characterized, underscoring the need for further research to clarify microbial contributions to civet coffee fermentation.

In the metabolomics analysis, the identified metabolites from fecal samples were subjected to scaling prior to unsupervised and supervised multivariate classification. Among the 12 metabolites that differed between the two diets prior to FDR correction, 6-hydroxyangolensic acid methyl ester, 4-aminobenzoic acid, and caffeine were associated with the coffee-cherry diet. This may be explained by the digestion and biotransformation of coffee pulp components, which is rich in carbohydrates and dietary fiber and also contains notable amounts of protein, lipids, caffeine, tannins, and polyphenols (Colodel, Reichembach & Petkowicz, 2023). Several negatively correlated compounds have documented antimicrobial properties. Caffeine, a major alkaloid in coffee cherries, has demonstrated inhibitory activity against multiple microorganisms *in vitro*, including *Escherichia coli* and *Salmonella enteritidis* (Gummadi, Bhavya & Ashok, 2012; McConnell & Bakermans, 2023). However, dietary coffee supplementation in murine models has produced mixed outcomes, with both increases and decreases in different microbial taxa reported (Nakayama & Oishi, 2013), highlighting the complexity of host–microbe responses to coffee-derived compounds. In the present study, caffeine was negatively associated with *Escherichia–Shigella, Enterococcus*, and *Mammaliicoccus*, consistent with its documented bacteriostatic effects.

Similarly, negative correlations were detected between 4-aminobenzoic acid, 6-hydroxyangolensic acid methyl ester, and the same bacterial genera. 4-aminobenzoic acid is synthesized by certain symbiotic bacteria and also obtained from dietary sources (Krátký et al., 2019). It has exhibited antibacterial activity against *Listeria monocytogenes*, *Salmonella enteritidis*, and *E. coli* (Richards, Xing & King, 1995). Meanwhile, 6-hydroxyangolensic acid methyl ester is a limonoid-like plant metabolite. Limonoids such as limonin have shown notable bacteriostatic effects against a range of bacteria and fungi (Fan et al., 2019). Although direct evidence for the antimicrobial activity of 6-hydroxyangolensic acid methyl ester remains limited, it is plausible that it exerts similar inhibitory effects, contributing to the reduced abundance of these taxa.

In contrast, several metabolites displayed positive correlations with microbial communities. These included plant-derived triterpenoids (soyasapogenols), bile acid conjugates (taurocholic acid and tauro-β-muricholic acid), lipid-related metabolites (LPE 16:0 isomers and lauryldiethanolamine), as well as hypotaurine (a taurine precursor) and 24-epimakisterone A (an ecdysteroid). Soyasaponin Bb and soyasapogenol B derivatives have been shown to modulate microbial composition in plant-associated systems by enriching specific bacterial taxa (Fujimatsu et al., 2020; Guang et al., 2014), suggesting that these triterpenoids may selectively shape microbial communities. Conjugated bile acids such as taurocholic acid and tauro-β-muricholic acid are closely linked to gut microbes involved in bile acid metabolism, and their availability can promote the expansion of certain bacterial groups (Devkota et al., 2012). Although some co-occurring metabolites lack direct mechanistic evidence linking them to the observed microbial shifts, their consistent association patterns suggest coordinated metabolic–microbial interactions. Further targeted experiments will be necessary to clarify whether these metabolites act as growth substrates, signaling molecules, or ecological modulators within the civet gut environment.

In addition to metagenomic profiling, unsupervised and supervised classification models were constructed to evaluate the metabolomic differences between the two diets. Both dietary regimens generated distinct metabolomic signatures, with most samples clustering according to diet. Nevertheless, several limitations in this pilot study warrant careful consideration. The relatively small sample size constrains the generalizability of the observed microbial and metabolomic patterns, and the loss of statistical significance after multiple testing correction underscores the need for larger cohorts to validate candidate biomarkers. Although a higher prevalence of caffeine was observed in excrete samples of civets feeding on a coffee-cherry diet, functional roles of other identified taxa remain largely inferred from related studies rather than directly demonstrated in civet systems. Future work combining metagenomics, transcriptomics, metabolomics, and controlled fermentation experiments would be valuable to clarify the metabolic pathways involved, particularly the contributions of the common microorganisms to coffee bean fermentation. Such investigations not only deepen the understanding of civet coffee fermentation but also inform biotechnological approaches for producing high-value coffee with reduced reliance on animal-derived processes.

## Conclusions

In summary, the integrated metagenomic and metabolomic analyses of civet fecal samples revealed that civets consuming coffee cherries harbored a more diverse bacterial community compared to those on a normal diet. This finding does not necessarily suggest that coffee cherry consumption promotes a healthier gut but rather indicates the potential roles of specific microorganisms in coffee fermentation within the civet gut microbiome. A marked shift in microbial composition was also observed between the two dietary groups, underscoring the strong influence of diet on gastrointestinal microbiota activity. Similarly, the metabolomic profiles demonstrated a clear separation between diets, with strong correlations detected between particular metabolites and the diet-associated metagenome. Nevertheless, the limited sample size constrains the generalizability of these findings, highlighting the need for larger-scale studies with extended sampling and longitudinal monitoring. Collectively, our findings reveal distinct gut microbiota profiles and fecal metabolite compositions in farmed civets in Vietnam, which differ from those reported in wild civets inhabiting coffee plantations in Malaysia (Watanabe et al., 2020) as well as from studies analyzing metabolites in Luwak coffee rather than fecal samples (Farag et al., 2023). To our knowledge, this is the first study to systematically investigate the effects of dietary shifts on both gut microbiota and fecal metabolites in civets.

## Supplemental Information

10.7717/peerj.21262/supp-1 Supplemental Information 1 Rarefaction curves illustrate the number of amplicon sequence variants (ASVs) detected at different sequencing depths. Each curve corresponds to an individual sample. Samples labeled T1 represent the coffee-cherry diet, whereas those labeled T2 indicate t.

10.7717/peerj.21262/supp-2 Supplemental Information 2 Relative abundance (%) of bacterial taxa at the phylum (A), family (B), and genus (C) levels in fecal samples from Asian palm civets fed either a coffee-cherry or a normal diet. Taxa with relative abundance exceeding 1% are displayed. All remaining taxa,.

10.7717/peerj.21262/supp-3 Supplemental Information 3 Number of raw reads and taxonomy assigned quality reads per sample.

10.7717/peerj.21262/supp-4 Supplemental Information 4 Significantly different bacterial taxa between Asian palm civets consuming coffee cherries and normal diet.

10.7717/peerj.21262/supp-5 Supplemental Information 5 List of identified metabolites detected in the fecal samples of Asian palm civets consuming coffee cherries and normal diet.

10.7717/peerj.21262/supp-6 Supplemental Information 6 Significantly different metabolites between Asian palm civets consuming coffee cherries and normal diet.

10.7717/peerj.21262/supp-7 Supplemental Information 7 Spearman’s correlation analysis of metabolites and bacterial genera.

## Additional Information and Declarations

### Field Study Permissions

The following information was supplied relating to field study approvals (*i.e*., approving body and any reference numbers):

Civet fecal samples used in this study were sourced from Kien Cuong Civet Coffee Company in Buon Ma Thuot City, Dak Lak Province, Vietnam, which operates under business registration certificate No. 6000771878 (issued on June 14, 2021, by the Department of Planning and Investment of Dak Lak Province, Vietnam). The care and management of palm civets at the company are conducted in cooperation with the Dak Lak Provincial Forest Protection Department, Vietnam.

### Data Availability

The following information was supplied regarding data availability:

The 16S rRNA sequencing data of civet’s fecal samples are available at NCBI: PRJNA1308768.

The metabolomics data is available at NIH Common Fund’s National Metabolomics Data Repository (NMDR), the Metabolomics Workbench Project ID PR002680; Project DOI: https://doi.org/10.21228/M87G0W.
