# Supplementary figures and images for: Metagenomic and metabolomic analyses of fecal samples from civet-digested coffee in Vietnam
Source: PeerJ. 2026 May 27;14:e21262. doi: 10.7717/peerj.21262 (PMC13221992; doi:10.7717/peerj.21262)

ASVs

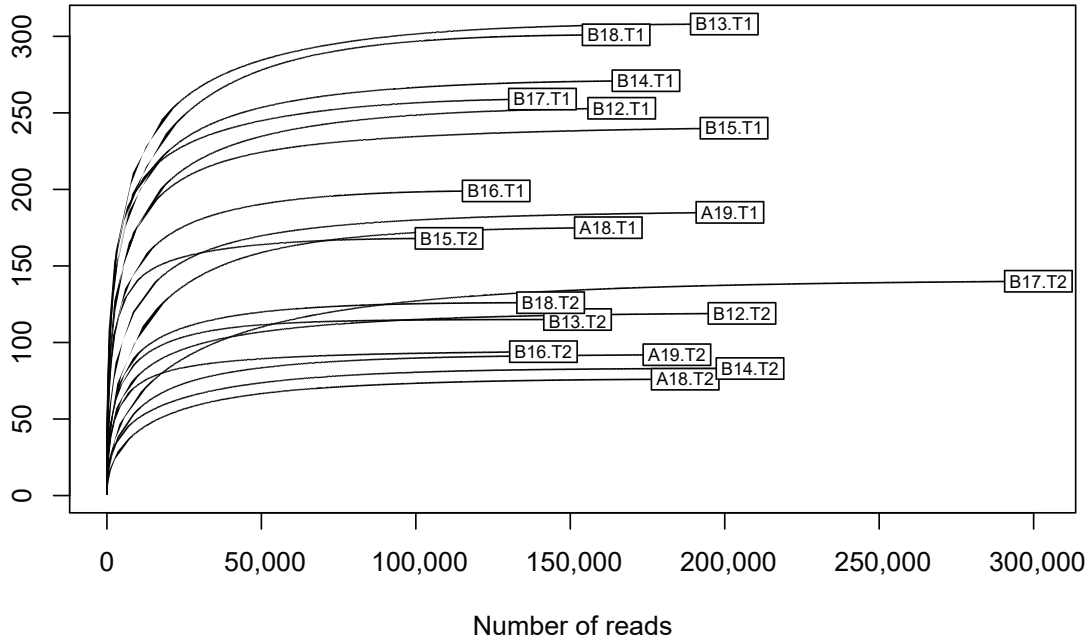

Supplement: Supplemental Information 1 [file peerj-14-21262-s001.pdf]

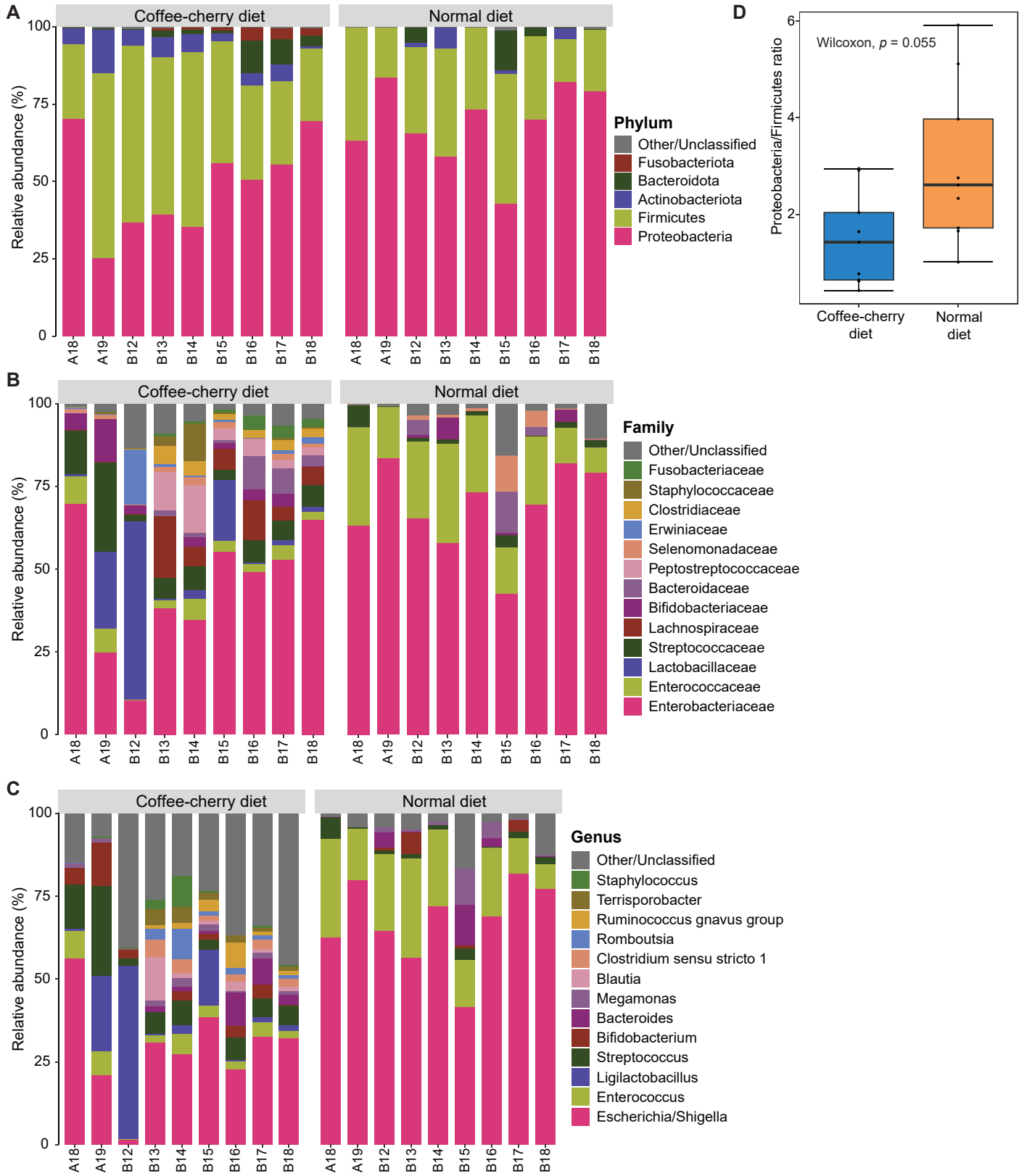

Supplement: Supplemental Information 2 [file peerj-14-21262-s002.pdf]
